# *Arabidopsis thaliana* as a suitable model host for research on interactions between plant and foliar nematodes, parasites of plant shoot

**DOI:** 10.1038/srep38286

**Published:** 2016-12-02

**Authors:** Dong-Wei Wang, Xiao-Fang Peng, Hui Xie, Chun-Ling Xu, De-Qiang Cheng, Jun-Yi Li, Wen-Jia Wu, Ke Wang

**Affiliations:** 1 Laboratory of Plant Nematology and Research Center of Nematodes of Plant Quarantine, Department of Plant Pathology, College of Agriculture, South China Agricultural University, Guangzhou, People’s Republic of China; 2 Center for Disease Control and Prevention of Guangdong Province, Guangzhou, People’s Republic of China

## Abstract

The rice white tip nematode (RWTN), *Aphelenchoides besseyi* and the chrysanthemum foliar nematode (CFN), *Aphelenchoides ritzemabosi* are migratory plant parasitic nematodes that infect the aboveground parts of plants. In this research, *Arabidopsis thaliana* was infected by RWTN and CFN under indoor aseptic cultivation, and the nematodes caused recognizable symptoms in the leaves. Furthermore, RWTN and CFN completed their life cycles and proliferated. Therefore, *A. thaliana* was identified as a new host of RWTN and CFN. The optimum inoculum concentration for RWTN and CFN was 100 nematodes/plantlet, and the optimum inoculum times were 21 and 24 days, respectively. For different RWTN populations, the pathogenicity and reproduction rates were different in the *A. thaliana* Col-0 ecotype and were positively correlated. The optimum *A. thaliana* ecotypes were Col-0 and WS, which were the most susceptible to RWTN and CFN, respectively. Additionally, RWTN was ectoparasitic and CFN was ecto- and endoparasitic in *A. thaliana*. The RWTN and CFN migrated from inoculated leaves to the entire plantlet, and the number of nematodes in different parts of *A. thaliana* was not correlated with distance from the inoculum point. This is a detailed study of the behavior and infection process of foliar nematodes on *A. thaliana*.

The rice white tip nematode (RWTN), *Aphelenchoides besseyi* Christie, 1942, and the chrysanthemum foliar nematode (CFN), *A. ritzemabosi* (Schwartz, 1911) Steiner and Buhrer, 1932, are migratory ecto- and endoparasites of leaves, buds, and/or other aboveground plant parts; therefore, these nematodes are commonly termed foliar/leaf and/or bud nematodes. RWTN has a host range of more than 200 plant species in 35 genera and is widely distributed in the primary rice growing areas of the world, with *Oryza sativa* L. as its primary host^1^,^2^,^3^. RWTN causes white tip disease and contributes significantly to estimated global annual losses of approximately $US16 billion^4^, and RWTN is listed in the top 10 plant-parasitic nematodes for economic impacts^5^. CFN is the most economically important foliar nematode species in chrysanthemum and is commonly called the chrysanthemum nematode. CFN has a host range of almost 200 different plant species, including ornamentals, crop plants and grasses^6^,^7^. Both species of nematode severely damage strawberry and also feed on some fungi^7^,^8^,^9^,^10^.

*Arabidopsis thaliana* has been an important model plant to study plant-plant pathogen interactions since the 1990s. Research using this model plant has deepened our understanding of the mechanisms of plant-pathogen interactions and allowed new concepts and methods for controlling plant diseases to be explored^11^,^12^,^13^,^14^. It had been reported that *A. thaliana* is a good host for several sedentary root-knot and cyst nematodes and two migratory nematodes, *Pratylenchus penetrans* and *Radopholus similis* which successfully infect and complete life cycles in *A. thaliana* under monoxenic conditions^11^,^15^. These nematodes are all endoparasites of underground parts of plants. Nematodes which parasitize in aboveground parts of plants, only *Bursaphelenchus xylophilus* and *B. mucronatus* were reported to infect *A. thaliana* through artificial wounds to cause similar initial infection symptoms as when pine tree are infected, however, these nematodes did not complete its life cycle and reproduce in *A. thaliana*^16^. To date, it is not reported that *A. thaliana* is a good host for nematodes parasitizing in aboveground parts of plant and there is also no detailed study of the behavior and infection process of these nematodes on *A. thaliana*, based on our understanding. The goals of this research were to demonstrate that *A. thaliana* was a good host for plant parasitic nematodes living in aboveground parts of plants and to establish a model system for studying the interaction between these nematodes and plants. The optimum inoculum conditions were determined, and a model system for the study of interactions between *A. thaliana* and two foliar nematodes was established.

## Results

### Symptoms and parasitism of RWTN and CFN in *A. thaliana*

**Figure 1:**
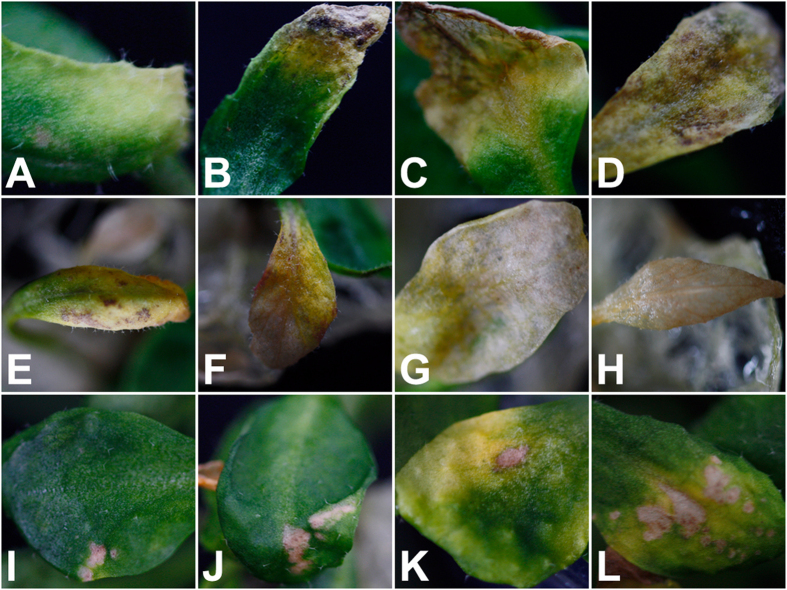
Symptoms on leaves of *Arabidopsis thaliana* Col-0 ecotype caused by *Aphelenchoides besseyi* and *A. ritzemabosi* infection. (**A**,**E** and **I**) early symptoms of leaves inoculated with nematodes from Ab-S24 and Ab-XI populations of *A. besseyi* and CFN population of *A. ritzemabosi* for 10 days. (**B**–**D**,**F**–**H** and **J**,**K**) development of symptoms in leaves inoculated with nematodes from Ab-S24 and Ab-XI populations of *A. besseyi* and CFN population of *A. ritzemabosi* at 14, 18 and 21 days, respectively. A and I were rated 1, B was rated 2, L was rated 3, C and K were rated 4, D-H were rated 5.

After the leaves of *A. thaliana* Col-0 ecotype were inoculated with RWTN from Ab-S24 and Ab-XI populations and the CFN population for 10–21 days, more nematodes were extracted from *A. thaliana* than were added in the inoculum. Additionally, *A. thaliana* showed different degrees of symptom severity (Fig. 1). Symptoms in leaves were observed recognizably after inoculation with RWTN. In leaves inoculated with Ab-S24 nematodes, the early symptoms appeared as chlorotic patches, which turned brown and then extended from leaf tips to petioles with time, with the ultimate result a yellowing of the entire leaf (Fig. 1A–D). In leaves inoculated with Ab-XI nematodes, symptoms were fading and yellowing from the margin of the leaf that spread gradually to the middle of veins and appeared as irregular brown spots that subsequently expanded to the entire leaf, which progressed from yellow to white and was finally hyalinised (Fig. 1E–H). After inoculation with CFN, initial symptoms in leaves were not observed; however, the inoculated leaves faded and turned brown in middle-late stages and were gradually covered with discontinuous dry and necrotic spots. The surrounding tissues yellowed, and the lesions enlarged with an increase in time (Fig. 1I–L). Thus, RWTN and CFN infected *A. thaliana* and the typical symptoms of leaves were a yellowing and brown spots, which became more serious through time.

**Figure 2:**
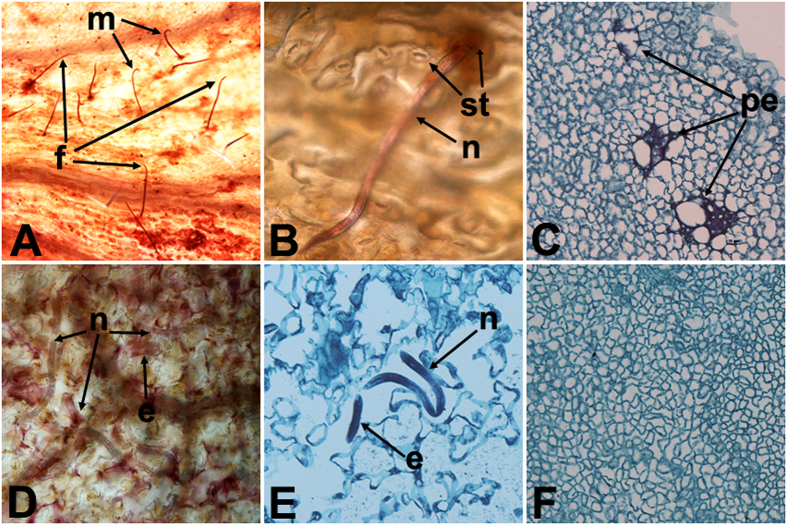
Histological sections of *Arabidopsis thaliana* Col-0 ecotype inoculated with *Aphelenchoides besseyi* and *A. ritzemabosi*. (**A**,**B**) *A. besseyi* in leaves; (**C**) perforation (pe) inside a leaf inoculated with *A. besseyi*; (**D**,**E**) *A. ritzemabosi* in leaves; (**F**) normal leaf; e: egg; f: female; m: male; n: nematode; st: stomata.

The primary infective stages of RWTN and CFN were mature females and males. In the leaves of *A. thaliana* infected with RWTN, the anterior region of nematodes was inserted into leaf tissues through leaf stomata, with the rest of the body outside the leaf (Fig. 2A,B). Some perforations occurred in the leaf and the margins browned, but no nematodes were found in the leaf tissues (Fig. 2C). In the leaves infected by CFN, the anterior region of nematodes was inserted into leaf tissue or entire bodies penetrated (Fig. 2D). The mesophyll cells of leaves were apparently broken and nematodes were found in the leaf tissues (Fig. 2E,F). Therefore, RWTN was ectoparasitic and CFN was ecto- and endoparasitic in *A. thaliana*.

### Effects of nematode inoculum concentration and infection time on symptoms in *A. thaliana*

**Figure 3:**
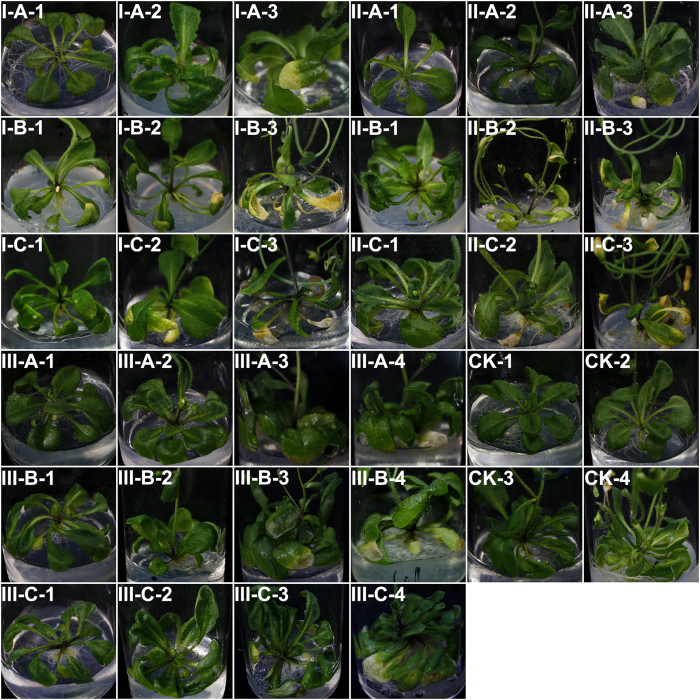
Symptoms on *Arabidopsis thaliana* Col-0 ecotype caused by *Aphelenchoides besseyi* and *A. ritzemabosi* infection. I, II and III: symptoms caused by nematodes from Ab-S24 and Ab-XI populations of *A. besseyi* and CFN population of *A. ritzemabosi*. (**A**–**C**) inoculum concentrations of 50, 100 and 150 nematodes per seedling; CK: blank control; 1–4. inoculation times were 10, 14, 21 and 24 days, respectively.

*Arabidopsis thaliana* Col-0 ecotype showed different degrees of symptom severity (Fig. 3 and Table 1) when leaves were inoculated with RWTN from Ab-S24 and Ab-XI populations and CFN population with 50,100 and 150 nematodes for 10–24 days. No symptoms were observed in *A. thaliana* inoculated with 50 nematodes from the three populations at 10 and 14 days after inoculation (DAI), inoculated with 100 and 150 nematodes from Ab-XI population at 10 DAI and with 100 and 150 from CFN population at 10 and 14 DAI. The symptom severity was rated 5 when inoculated with 100 and 150 nematodes of Ab-S24 population and with 150 nematodes of Ab-XI population at 21 DAI. The symptom severity of *A. thaliana* inoculated with 150 nematodes and 100 nematodes of CFN population at 24 DAI, with 150 nematodes of Ab-XI population at 14 DAI, with 100 nematodes of Ab-XI population at 21 DAI and with 150 nematodes of CFN population at 21 DAI were gradually decreased. No significant differences among these eight treatments (P > 0.05). The symptoms severity of the three treatments rated 5 were significantly higher than the remaining ten treatments (P < 0.05), the symptoms severity of other five treatments of the eight treatments were also significantly higher than the remaining treatments (P < 0.05) exception for the treatment inoculated with 50 nematodes of CFN population at 24 DAI. In the remaining ten treatments, the symptoms severity of the treatment inoculated with 50 nematodes of CFN population at 24 DAI were significantly higher than the treatments inoculated with 100 nematodes of Ab-S24 population at 10 DAI and with 50 nematodes of CFN population at 21 DAI (P < 0.05), no significant differences among the other treatments (P > 0.05).

**Figure 4:**
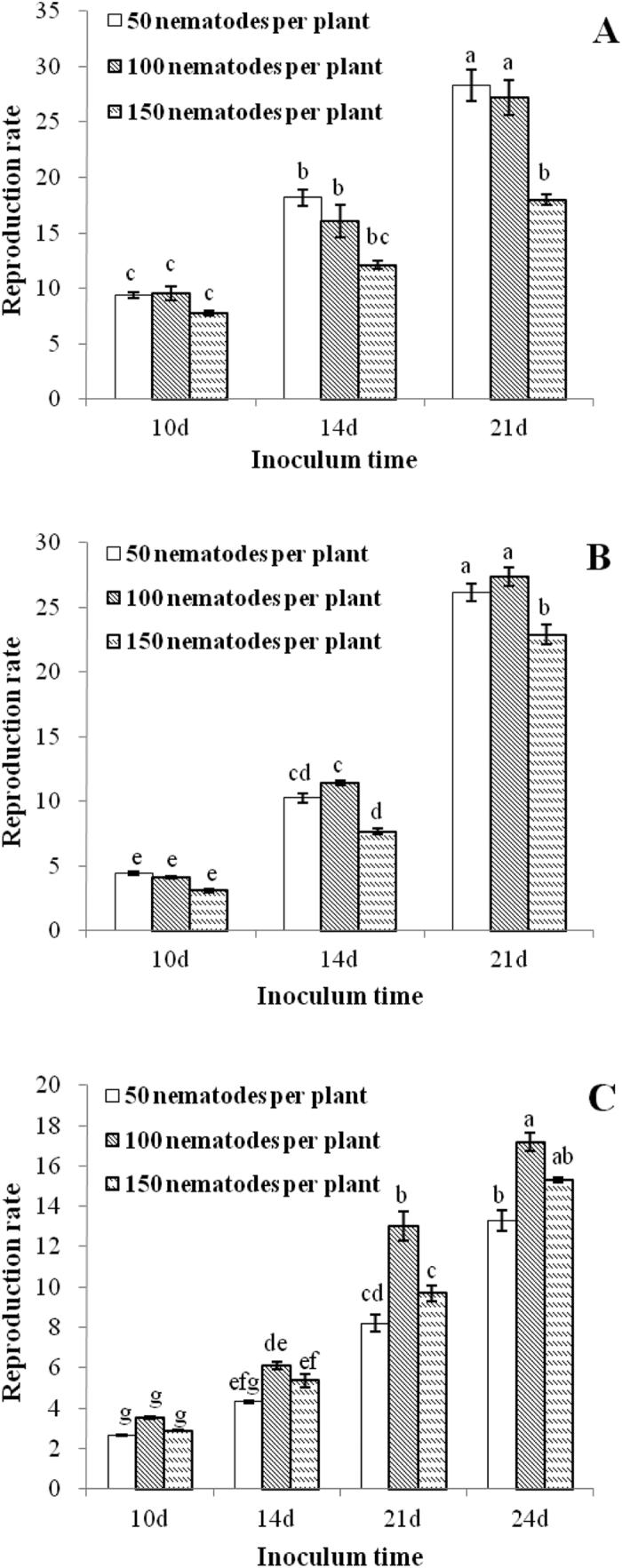
Reproduction rates (mean ± SE; n = 6) of *Aphelenchoides besseyi* and *A. ritzemabosi* in *Arabidopsis thaliana* Col-0 ecotype. (**A**–**C**) nematodes from Ab-S24 and Ab-XI populations of *A. besseyi* and CFN population of *A. ritzemabosi*. Identical lower case letters indicate means are not significantly different (P > 0.05) for different inoculation times according to Duncan’s Multiple Range Test.

At the identical inoculum concentration, the reproduction rates of RWTN from Ab-S24 and Ab-XI populations and CFN population increased significantly (P < 0.05) with the increase in time since inoculation (P < 0.05), except for the inoculation with 150 nematodes from Ab-S24 population between 10 and 14 days and the inoculations with 50 nematodes of CFN population after 10 and 14 days (Fig. 4). At 10 DAI, the reproduction rates of Ab-S24 and Ab-Xi nematodes for the three concentrations of inoculum were 9.41, 9.54, and 7.78 and 4.40, 4.13, and 3.10, respectively, with no significant differences (P > 0.05) among the three inoculum concentrations for each population. At 14 DAI, the reproduction rate of Ab-S24 nematodes was significantly different (P < 0.05) between inoculations of 50 and 150 nematodes, with no significant differences (P > 0.05) among other treatments. The maximum reproduction rate of Ab-Xi nematodes was 11.42 when inoculated with 100 nematodes, which was significantly higher (P < 0.05) than the inoculation with 150 nematodes. The difference in rates was not significant (P > 0.05) between inoculations with 50 and 100 nematodes. The maximum reproduction rate of CFN nematodes was 3.50 and 6.10 at 10 and 14 DAI, respectively, when inoculated with 100 nematodes, with no significant differences (P > 0.05) among the three levels of inoculums. At 21 DAI, the reproduction rates of Ab-S24 and Ab-Xi nematodes for the three inoculum concentrations were 28.33, 27.23 and 17.98 and 26.18, 27.35 and 22.90, respectively. The difference in rates was not significant (P > 0.05) between 50 and 100 nematodes for either population; however, rates were significantly higher (P < 0.05) than those of leaves inoculated with 150 nematodes. The maximum reproduction rate of CFN nematodes was 12.99 for the inoculation with 100 nematodes at 21 DAI, which was significantly higher (P < 0.05) than the rate after inoculation with 50 and 150 nematodes. The difference was not significant (P > 0.05) between inoculations with 50 and 150 nematodes. At 24 DAI, the maximum reproduction rate of CFN nematodes was 17.15 when inoculated with 100 nematodes, with no significant difference (P > 0.05) between inoculations with 100 and 150 nematodes, but these rates were significantly higher (P < 0.05) than the rate with inoculation of 50 nematodes.

Therefore, to test pathogenicity of RWTN and CFN to *A. thaliana*, the appropriate inoculum concentration was 100 or 150 nematodes, with the initial symptoms observed in leaves inoculated for 10 or 14 days and 21 days, respectively, the degree of symptom severity determined at 21 and 24 DAI, respectively. To test the reproduction rate of RWTN and CFN in *A. thaliana*, the appropriate inoculum concentration was 50 or 100 nematodes and 100 nematodes, respectively. With consideration of symptoms in leaves and reproductive rates, the optimal inoculum concentration of RWTN and CFN was 100 nematodes per seedling.

### Susceptibility of *A. thaliana* ecotypes to RWTN and CFN

The symptoms in Col-0, Ws, Ler, Sha and Chi ecotypes of *A. thaliana* were different after inoculation with 100 RWTN from populations Ab-S24 and Ab-XI for 10-21 days (see Supplementary Fig. S1). After inoculation with the two populations for 10 days, the five ecotypes did not show symptoms. After inoculation with Ab-S24 nematodes for 14 days, symptoms were observed in inoculated leaves, with the worst symptoms in ecotype Col-0, which had inoculated leaves that were yellow from the tip to the middle of the leaf. The inoculated and adjacent non-inoculated leaves of Chi ecotype yellowed, and the area of yellowing was larger than that in the three other ecotypes. Leaf spots were observed in inoculated and adjacent non-inoculated leaves of ecotype Ler, and the leaves of this ecotype shrank. In WS and Sha ecotypes, inoculated leaves yellowed slightly. After inoculation with Ab-XI nematodes for 14 days, the tips of inoculated leaves of four ecotypes, with Chi ecotype the exception, yellowed. After inoculation with the two populations for 21 days, symptoms were observed in the five ecotypes. The symptoms in Col-0 ecotype were the most severe; these plants had fewer and small leaves, the inoculated leaves were completely browned, and the adjacent non-inoculated leaves showed obvious yellowing. The symptoms of the other four ecotypes inoculated with Ab-XI nematodes were similar, with inoculated leaves yellowed from the leaf margins and with shrinkage of leaves. The leaf tips of Chi ecotype inoculated with Ab-S24 nematodes yellowed, with many spots browning in the middle of leaves and some adjacent non-inoculated leaves that yellowed, and the symptoms in Ler and Sha ecotypes were middle of veins that were browned and margins of inoculated leaves that were yellowed, with the undersides of adjacent non-inoculated leaves obviously yellowed. The symptoms of Ws ecotype were the least severe, with inoculated leaves that yellowed. Therefore, the symptoms in Col-0 ecotype were the most severe among the five ecotypes inoculated with RWTN.

**Figure 5:**
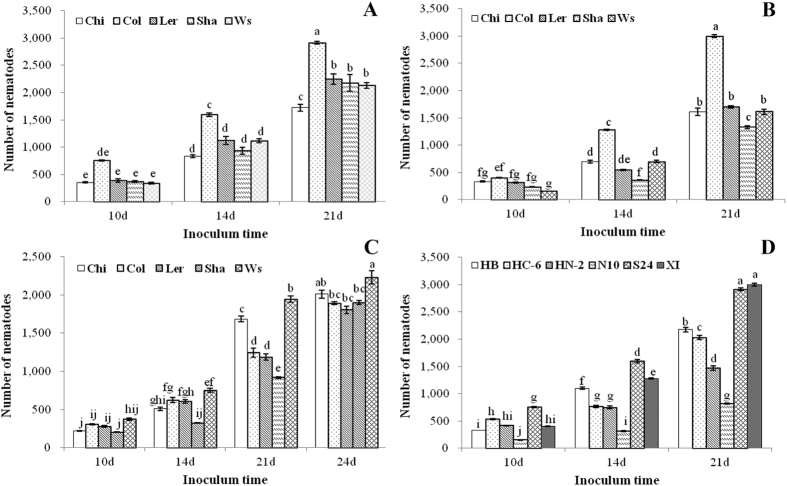
Number reproduced (mean ± SE; n = 6) of *Aphelenchoides besseyi* and *A. ritzemabosi* in *Arabidopsis thaliana*. (**A**–**C**) nematodes from Ab-S24 and Ab-XI populations of *A. besseyi* and CFN population of *A. ritzemabosi* five *A. thaliana* ecotypes after inoculation with 100 nematodes. (**D**) nematodes from six *A. besseyi* populations in *A. thaliana* Col-0 ecotype inoculated with 100 nematodes for 10–21 days. Identical lower case letters indicate means are not significantly different (P > 0.05) for different inoculation times according to Duncan’s Multiple Range Test.

The reproduction number of RWTN from two populations inoculated in the five ecotypes with 100 nematodes for 10–21 days were higher than one in all treatments. The numbers of Ab-S24 and Ab-XI nematodes increased significantly (P < 0.05) with the increase in time since inoculation in the identical ecotype, with the exception of Ab-XI nematodes inoculated in the Sha ecotype for 10 and 14 days (Fig. 5A,B). At 10 DAI, the maximum number of Ab-S24 nematodes after inoculation in Col-0 ecotype was 755.17, which was significantly higher (P < 0.05) than that in the other ecotypes, with no significant differences (P > 0.05) among the other ecotypes. The maximum number of Ab-XI nematodes was 399.83 when inoculated in Col-0 ecotype, which was significantly higher (P < 0.05) than that in Sha and WS ecotypes. The minimum number was 152.00 when inoculated in Ws ecotype, which was significantly lower (P < 0.05) than that in the other ecotypes, except for Sha ecotype. No significant differences were observed among Chi, Ler and Sha ecotypes. At 14 DAI, the maximum number of Ab-S24 and Ab-XI nematodes was 1598.17 and 1278.67, respectively, in Col-0 ecotype, which was significantly higher (P < 0.05) than that in other ecotypes. No significant differences (P > 0.05) were observed among the other ecotypes inoculated with Ab-S24 nematodes. The minimum number of Ab-XI nematodes was 359.50 when inoculated in Sha ecotype, which was significantly lower (P < 0.05) than that in other ecotypes. The number of Ab-XI nematodes was not significantly different (P > 0.05) between Chi and Ws ecotypes but was significantly higher (P < 0.05) than that in Ler ecotype. At 21 DAI, the maximum number of Ab-S24 and Ab-XI nematodes was 2913.33 and 2996.67, respectively, when inoculated in Col-0 ecotype, which was significantly higher (P < 0.05) than that in other ecotypes. The minimum number of Ab-S24 nematodes was 1721.00 with inoculation in Chi ecotype, which was significantly lower (P < 0.05) than that in other ecotypes. The number of nematodes was not significantly different (P > 0.05) among Ler, Sha and Ws ecotypes. The minimum number of Ab-XI nematodes was 1329.33 when inoculated in Sha ecotype, which was significantly lower (P < 0.05) than that in other ecotypes. The number of nematodes was not significantly different (P > 0.05) among Chi, Ler and Ws ecotypes. Therefore, based on the severity of symptoms and nematode reproduction, *A. thaliana* Col-0 ecotype was the most susceptible to RWTN, followed by Ler and WS ecotypes, and then Chi and Sha ecotypes.

Following inoculation of *A. thaliana* with CFN for 10 and 14 days, no symptoms were observed on the five ecotypes (see Supplementary Fig. S1). At 21 DAI, the most severe symptoms were on Ws ecotype, with brown spots and yellowing of inoculated leaves and obvious yellowing of adjacent non-inoculated leaves. In the other four ecotypes, the tips and margins of inoculated and adjacent non-inoculated leaves were yellow. At 24 DAI, the symptoms were further aggravated; the most severe symptoms remained on Ws ecotype, with many leaves in lower parts of the plant that were completely yellow and with parts of leaf tips that whitened. Chi ecotype had less severe symptoms, with leaves of the entire plant that were slight and curling and with yellowish-brown between veins. The area of yellowing leaves of Ler ecotype was larger than that of Col-0 and Sha ecotypes, and this ecotype had inoculated leaves that were completely yellow and with brown spots that spread to many leaves. Therefore, symptoms of CFN were the most severe in leaves of Ws ecotype, followed by Chi and Ler ecotypes, and then the Col-0 and Sha ecotypes.

The reproduction number of CFN inoculated in the five *A. thaliana* ecotypes with 100 nematodes for 10–21 days were higher than a value of one in all treatments, and the number of CFN increased significantly (P < 0.05) with the increase in time since inoculation for the identical ecotype, except for Sha ecotype when inoculated for 10 and 14 days (Fig. 5C). At 10 DAI, the number of nematodes was not significantly different (P > 0.05) among the five *A. thaliana* ecotypes. At 14 DAI, the number of CFN was 752.67, 625.50, 603.50, 509.17 and 323.33 in Ws, Col-0, Ler, Chi, and Sha ecotypes, respectively. The number of nematodes in WS ecotype was significantly higher (P < 0.05) than that in Chi and Sha ecotypes, with no significant difference between Chi and Sha ecotypes. At 21 DAI, the number of CFN was 1946.17, 1687.50, 1245.83, 1189.83 and 918.33 in Ws, Col-0, Ler, Chi, and Sha ecotypes, respectively. No significant difference (P > 0.05) was detected between Col-0 and Ler ecotypes, but significant differences (P < 0.05) were detected among other treatments. At 24 DAI, the maximum number of nematodes was 2232.33 in Ws ecotype, which was significantly higher (P < 0.05) than that in other ecotypes. The minimum number of nematodes was 1860.33 in Ler ecotype, which was significantly lower (P < 0.05) than that in Chi ecotype, with no significant differences (P > 0.05) among Col-0, Ler and Sha ecotypes. Therefore, based on the severity of symptoms and nematode reproduction, the Ws ecotype of *A. thaliana* was the most susceptible to CFN, followed by Chi, Ler, Col-0 and Sha ecotypes.

### The pathogenicity of six RWTN populations to *A. thaliana*

After inoculation of *A. thaliana* Col-0 ecotype with RWTN from six populations (Supplementary Table S1) for 10 days, no symptoms were observed on the leaves, with the exception of yellowing of leaf tips of leaves inoculated with Ab-S24 nematodes (see Supplementary Fig. S2). At 14 DAI, the leaves inoculated with Ab-S24 nematodes were completely yellow. The tips of leaves inoculated with Ab-HB and Ab-XI nematodes were yellow, and for *A. thaliana* inoculated with Ab-XI nematodes, the leaves of entire plants were few and small and inoculated leaves were obviously yellow. Symptoms were not observed in leaves from the other three treatments. At 21 DAI, all inoculated leaves were yellowing; the most severe symptoms were in leaves inoculated with Ab-S24 and Ab-XI nematodes, and the leaves of entire plants were few and small, with inoculated leaves completely faded and hyalinised. The adjacent non-inoculated leaves were obviously yellowing, with brown spots appearing and with some leaves shrinking. The inoculated and adjacent non-inoculated leaves of plantlets inoculated with nematodes from the Ab-HC6 population were yellow; whereas the tips of leaves inoculated with Ab-HB and Ab-HN2 nematodes and the leaves inoculated with Ab-N10 nematodes were yellowing. When leaves of *A. thaliana* were inoculated with RWTN from six populations, different degrees of symptom severity were demonstrated; the symptoms of plantlets inoculated with Ab-S24 nematodes were the most severe, followed by those of Ab-XI nematodes, and the least severe symptoms were in plantlets inoculated with Ab-N10 nematodes.

The reproduction number of RWTN from six populations inoculated in *A. thaliana* Col-0 ecotype with 100 nematodes for 10–21 days were higher than one for all treatments, and the nematode number increased significantly (P < 0.05) with the increase in time since inoculation (Fig. 5D). At 10 DAI, the numbers of Ab-S24 and Ab-N10 nematodes were 755.83 and 152.50, respectively, which were significantly higher and lower (P < 0.05), respectively, than those of other populations. The number of Ab-HC6, Ab-HN2, Ab-XI and Ab-HB nematodes was 533.83, 413.83, 400.33 and 323.33, respectively, with no significant differences (P > 0.05) among Ab-HN2, Ab-XI and Ab-HB nematodes but with a significant difference (P < 0.05) between the three treatments and that of Ab-HB. At 14 DAI, the number of Ab-S24, Ab-XI, Ab-HB, Ab-HC6, Ab-HN2 and Ab-N10 nematodes was 1598.17, 1278.83, 1099.67, 765.50, 748.00 and 311.00, respectively, with no significant difference (P > 0.05) between Ab-HC6 and Ab-HN2 nematode numbers but with significant differences (P < 0.05) among the other treatments. At 21 DAI, the number of Ab-S24, Ab-XI, Ab-HB, Ab-HC6, Ab-HN2 and Ab-N10 nematodes was 2996.67, 2913.83, 2176.33, 2028.00, 1471.83 and 819.67, respectively, with no significant difference (P > 0.05) between Ab-XI and Ab-S24 nematode numbers but with significant differences (P < 0.05) among the other treatments.

Thus, the nematodes from six different RWTN populations infected and completed life cycles in *A. thaliana*. However, the number by reproduction was different for RWTN from six populations when inoculated in *A. thaliana* ecotype Col-0, with maximum reproduction of Ab-S24 and Ab-XI nematodes and minimum reproduction of Ab-N10 nematodes.

### Migration characteristics of RWTN from two populations and of CFN from one population in *A. thaliana*

**Figure 6:**
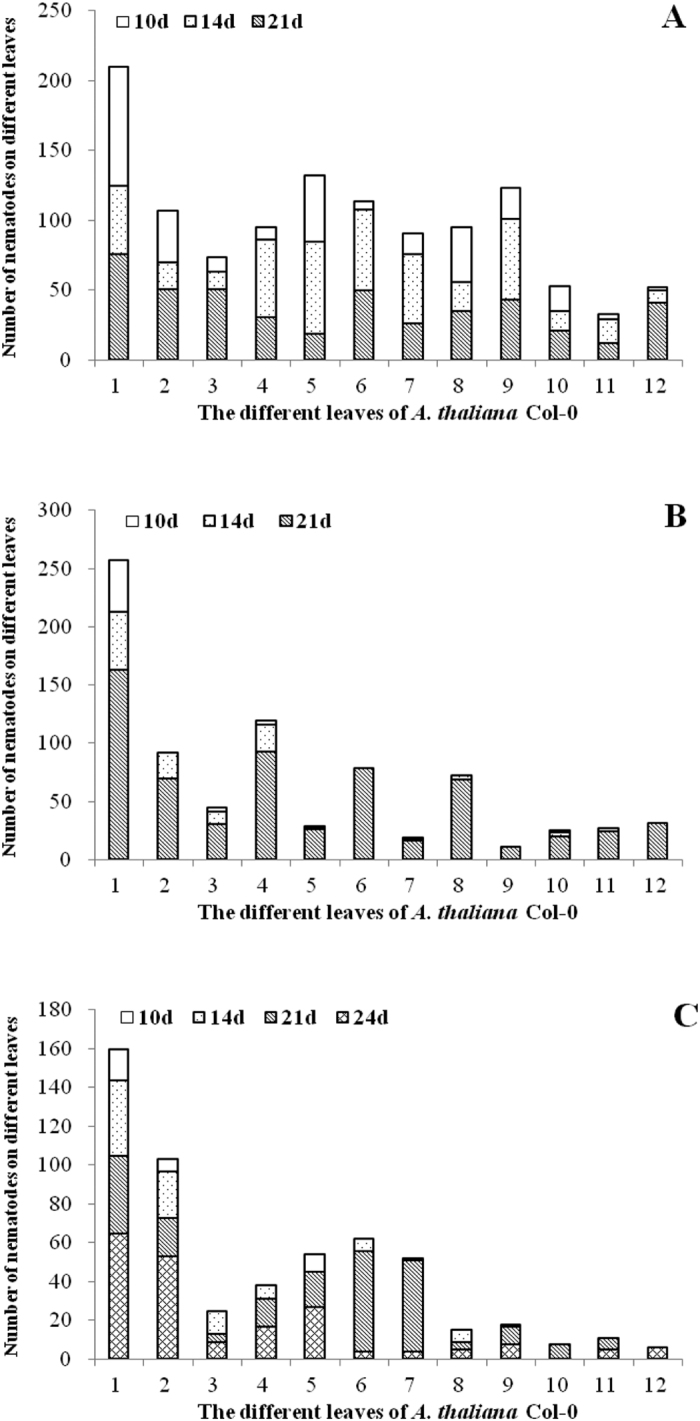
Number (mean ± SE; n = 6) of *Aphelenchoides besseyi* and *A. ritzemabosi* in different leaves of *Arabidopsis thaliana* Col-0 ecotypes. (**A**–**C**) nematodes from Ab-S24 and Ab-XI populations of *A. besseyi* and CFN population of *A. ritzemabosi*.

**Figure 7:**
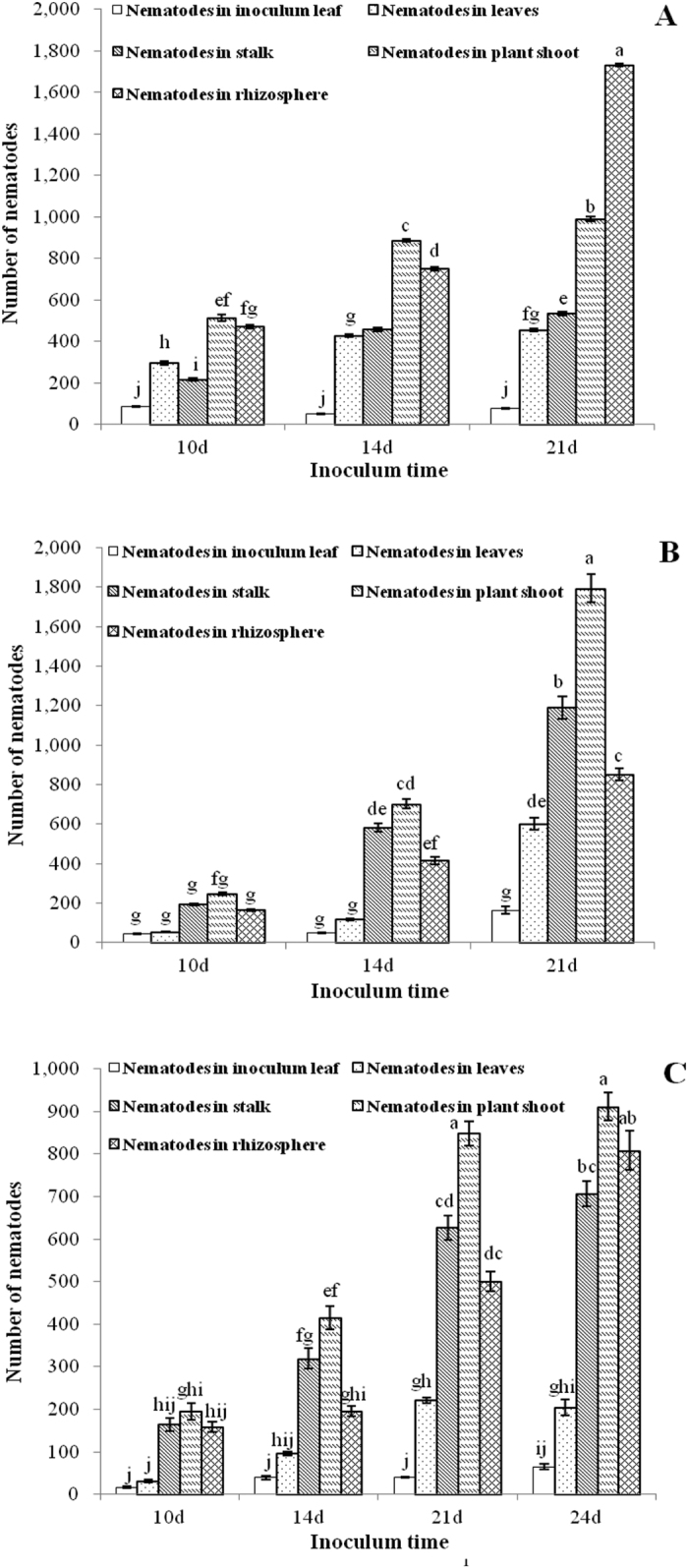
Number (mean ± SE; n = 6) of *Aphelenchoides besseyi* and *A. ritzemabosi* in different parts of *Arabidopsis thaliana* Col-0 ecotypes. (**A**–**C**) nematodes from Ab-S24 and Ab-XI populations of *A. besseyi* and CFN population of *A. ritzemabosi*. Identical lower case letters indicate means are not significantly different (P > 0.05) for different inoculation times according to Duncan’s Multiple Range Test.

Following inoculation of *A. thaliana* Col-0 ecotype with RWTN from Ab-S24 and Ab-XI populations and CFN from one population with 100 nematodes for 10–21 days, nematodes were in the inoculated leaf (the first leaf), non-inoculated leaves, stalk and rhizosphere of *A. thaliana* (Figs 6 and 7). The number of Ab-S24 nematodes was 49.50 in the inoculated leaves at 14 DAI, with the maximum and the minimum number of nematodes in non-inoculated leaves of 66 and 9, respectively. The number of CFN was 39.83 in inoculated leaves at 21 DAI, with the maximum and the minimum number of nematodes in non-inoculated leaves of 52 and 0, respectively. For the other treatments, the number of nematodes in inoculated leaves was more than that in non-inoculated leaves. The number of nematodes in non-inoculated leaves was not correlated with the distance from inoculated leaves. The number of Ab-S24 nematodes in leaves and stalk was 295.67 and 216.67, respectively, which were significantly different (P < 0.05) at 10 DAI. More nematodes were in stalks than in leaves for the other treatments; however, no significant differences (P > 0.05) were observed when inoculated with Ab-Xi nematodes for 10 days and with Ab-S24 nematodes for 14 days, although the differences were significant (P < 0.05) in the other treatments. At 21 DAI, the number of Ab-S24 nematodes in the rhizosphere and shoot of plant was 1731.83 and 989.50, respectively, with the difference significant (P < 0.05). The number of nematodes in plant shoots was greater than that in the rhizosphere in the other treatments. The differences were not significant (P > 0.05) between numbers in the rhizosphere and shoot among the treatments with inoculation of the three populations for 10 days and the CFN population inoculation for 24 days. However, the differences were significant (P < 0.05) among the other treatments.

Therefore, RWTN and CFN migrated from inoculated leaf to the entire plantlet, and the number of nematodes from different parts of *A. thaliana* was not correlated with distance from the inoculum point. Infection spots were observed only in the leaves, with no apparent infection points in the root and stalk of *A. thaliana*, despite the extraction of many nematodes from these plant parts. The eggs, larvae, females and males were observed in the rhizosphere medium, which indicated that RWTN and CFN completed their life cycles in the rhizosphere of *A. thaliana*.

## Discussion

In this research, *A. thaliana* was inoculated with RWTN and CFN under aseptic conditions, and these nematodes infected, completed life cycles and proliferated in *A. thaliana*; thus, *A. thaliana* is a new suitable host for RWTN and CFN. It has been reported that plant parasitic nematodes living in underground parts of plants, including root-knot, cyst, root-lesion and banana burrowing nematodes, can parasitize *A. thaliana* and complete life cycles^11^,^15^. However, it is not reported that plant parasitic nematodes living in aboveground parts of plant can parasitize *A. thaliana* and complete life cycles^16^. The RWTN and CFN are typical plant parasitic nematodes living in aboveground parts of plants, and this study is the first to report that these nematodes can parasitize and complete life cycles in *A. thaliana*. In addition, this study is the first detailed study of the behavior and infection process of foliar nematodes on *A. thaliana*. This research provides a new foundation to study the interactions between those nematodes parasitizing in aboveground parts of plants and the host *A. thaliana*.

This research confirmed that the rate of damage progression and the degree of symptom severity in *A. thaliana* were positively correlated with the inoculum concentrations. Although the reproduction rate was positively correlated with time since inoculum, the rate was not correlated with inoculum concentrations. Based on the results, to study the interaction between the two foliar nematodes and *A. thaliana*, the optimum inoculum concentrations of RWTN and CFN were both 100 nematodes/plantlet. Six RWTN populations showed pathogenicity to *A. thaliana*, but the damaging symptoms to leaves and the reproduction rates were different among the six populations. Additionally, the pathogenicity of an RWTN population was positively correlated with the reproduction rate. Of the six RWTN populations, nematodes from population Ab-S24 that were collected from *Fragaria ananassa* in Shenzhen, Guangdong, had the highest pathogenicity to *A. thaliana*; followed by nematodes from Ab-XI population collected from *O. sativa* in Xiamen, Fujian; and the pathogenicity of nematodes from the Ab-N10 population collected from *O. sativa* in Nanjing, Jiangsu, was the weakest. The severity of damage to rice caused by populations of RWTN from different hosts was different, with this difference influenced by the original host^17^. For example, the pathogenicity to rice of an RWTN population from strawberry was weaker than that of populations from rice, and the reproduction rate was not positively correlated with the pathogenicity of RWTN on rice. Those results are not consistent with our results, which may be because *A. thaliana* was not the original host for these nematodes; however, both studies showed that the pathogenicity of different RWTN populations was different on the identical host.

Five *A. thaliana* ecotypes were susceptible to RWTN and CFN populations, but the susceptibility differed among ecotypes. The *A. thaliana* Col-0 ecotype had the highest susceptibility to RWTN and Chi and Sha ecotypes had the lowest susceptibility. The WS and Sha ecotypes had the highest and the lowest susceptibility to CFN, respectively. Zhao *et al*. reported that *A. thaliana* Col-0 and Ler ecotypes have the highest susceptibility to *B. xylophilus* and *B. mucronatus*, respectively, among nine *A. thaliana* ecotypes^16^. Therefore, the susceptibility of *A. thaliana* to aphelench nematodes differed among *A. thaliana* ecotypes and between nematode species. To study the relationships between aphelenchs and their hosts, the best approach is to select the optimum ecotype among different *A. thaliana* ecotypes by testing the response to the parasitism and pathogenicity of different nematode populations.

RWTN was ectoparasitic and CFN was ecto- and endoparasitic in *A. thaliana*, which is consistent with the parasitic mode of RWTN on *O. sativa* and that of CFN on chrysanthemum^1^,^6^. The migration of RWTN and CFN is not related to gravity but that the distribution and migration of these nematodes are affected by humidity and roughness of stalk^18^,^19^. Wallace *et al*. reported that 90% of adult nematodes of CFN were distributed in the upper parts of host plant when water was sufficient, showing also a tendency against gravity^20^. Additionally, the RWTN that parasitizes rice and CFN do not survive in soils, whereas the RWTN that parasitizes strawberry does survive in soils^1^,^6^. The migration and distribution of RWTN and CFN that parasitized *A. thaliana* were not consistent with reports in the literature. The two foliar nematodes that parasitized *A. thaliana* in our work migrated from inoculated leaves to the entire plantlet and completed their life cycles in the medium of the plant rhizosphere; however, the symptoms were observed only in the leaves.

In conclusion, this study was the first to demonstrate that RWTN and CFN infected and parasitized *A. thaliana* and detailed study of the behavior and infection process of foliar nematodes on *A. thaliana*. Thus, *A. thaliana* was identified as a new host for RWTN and CFN, and RWTN was ectoparasitic and CFN was ecto- and endoparasitic. The parasitism and pathogenicity to *A. thaliana* were variation between two foliar nematodes and among RWTN populations. In this research, a new model system for studying the interactions between foliar nematodes and hosts was established using *A. thaliana*, and with this system, a new approach is provided to study the mechanisms of host-nematode interactions, which will lead to exploration of new methods for control of foliar nematodes.

## Methods

### Nematode cultivation and sterilization

Six RWTN populations and a CFN population (Supplementary Table S1) were cultured on excised carrot callus in Petri dishes (diameter 6 cm) at 25 °C in an incubator^17^,^21^. The nematodes were cultured for approximately 20 days on carrot callus. Nematodes were surface-sterilized in 0.01% chlorhexidine for 90 s and in 0.1% HgCl_2_ solution for 10 min and then washed five times in sterile distilled water^21^. To determine inoculum concentrations, sterilized nematodes were counted under a light microscope to prepare suspensions with 50, 100, 150 and 300 nematodes per 10 μL for use in subsequent experiments. The nematode inoculation tests were all conducted under aseptic conditions in *A. thaliana*.

### *A. thaliana* ecotypes and cultivation

Prof. Heng Jian (Department of Plant Pathology, China Agricultural University) kindly provided the ecotypes of *A. thaliana,* which included Chi, Col-0, Ler, Sha and Ws ecotypes. The cultivation of *A. thaliana* followed that of Hammes *et al*., with some improvements^22^. After *A. thaliana* germinated and grew to a stage with a centre leaf and four functional leaves (approximately a week after germination), plants were transferred under aseptic conditions into flat tubes (3×7 cm) that contained a 2-cm-thick layer of MS medium; the tubes were sealed with breathable membranes and fixed with rubber bands. The germination/growth assays were performed under a 16 hours light (28 °C)/8 hours dark (25 °C) cycle in an incubator.

### Pathogenicity

To study the pathogenicity and parasitism of RWTN and CFN in *A. thaliana* Col-0 ecotype, the first leaf of each seedling was inoculated with 300 mixed-stage nematodes from Ab-S24, Ab-XI and CFN populations. The leaves in *A. thaliana* were observed for symptoms 10, 14, 18 and 21 days after inoculation. Three inoculum concentrations and times of inoculation were evaluated to determine the effect of RWTN and CFN infection rate on *A. thaliana*. The first leaf was inoculated with 50, 100 or 150 mixed-stage nematodes from Ab-S24, Ab-XI or CFN populations. The leaves of *A. thaliana* were observed for symptoms and nematodes were extracted at 10, 14 and 21 DAI, and for the nematodes from the CFN population, the experiment was extended to 24 days because of the long reproductive cycle. The host preference was designated as followed: reproduction rate (Rf) > 1.0, host plants, 1 ≥ Rf > 0, poor hosts, and Rf = 0, non-host plants as described by Goo and Sipes^23^.

To study the pathogenicity of different RWTN populations to *A. thaliana*, 100 mixed-stage nematodes from six RWTN populations (Table 1) were used to inoculate the first leaf of *A. thaliana*. The leaves of *A. thaliana* were observed for symptoms and nematodes were extracted at 10, 14 and 21 DAI. The blank control in the pathogenicity test was treated identically except that sterile distilled water was used.

### *A. thaliana* ecotype screening

The ecotypes Chi, Col-0, Ler, Sha and Ws of *A. thaliana* were assessed for susceptibility to RWTN and CFN. The first leaf of the five *A. thaliana* ecotypes was inoculated with 100 mixed-stage nematodes from Ab-S24, Ab-XI and CFN populations. The leaves of *A. thaliana* were observed for symptoms and nematodes were extracted at 10, 14 and 21 DAI, and for the CFN population, the experiment was extended to 24 days.

### Migration of nematodes in *A. thaliana*

To evaluate the migration characteristics of RWTN and CFN in *A. thaliana*, the first leaf of *A. thaliana* Col-0 ecotype plants was inoculated with 100 mixed-stage nematodes from Ab-S24, Ab-XI and CFN populations. The leaves in *A. thaliana* were observed for symptoms, and nematodes in the medium, roots and aboveground parts of *A. thaliana* were extracted at 10, 14 and 21 DAI, and for the CFN population, the experiment was extended to 24 days.

### Symptom observations

Acording to Zhen *et al*.^24^, the rating of symptom severity on *A. thaliana* caused by foliar nematodes were assigned as follows: rated 0 = no lesion/chlorosis, 1 = 10% lesion/chlorosis, 2 = 11–25% lesion/chlorosis, 3 = 26–50% lesion/chlorosis, 4 = 51–75% lesion/chlorosis, 5 = 75% or more lesion/chlorosis. Symptomatic leaves were selected at random and stained with acid fuchsin according to Volvas *et al*.^25^. Leaves without symptoms and the control leaves were stained using the methods of Zhao *et al*.^16^. Paraffin sections were produced according to the description provided by Sasanelli *et al*.^26^. To confirm the parasitism of RWTN and CNF in *A. thaliana*, sections were examined on glass slides and photographed under a Nikon Eclipse 90i microscope.

### Nematode extraction

The leaves of *A. thaliana* were cut into 1 cm^2^ pieces and the stalks and shoots were cut into 1 cm fragments. These pieces and fragments were soaked in tap water for 48 h at room temperature, and the nematodes were collected using nested sieves of 0.147 mm and 0.026 mm and counted under a stereomicroscope. The MS medium was mashed with a blender and filtered through combined sieves to collect nematodes, and these nematodes were also counted under a stereomicroscope.

### Data analysis and processing

In this study, each treatment was replicated three times and each experiment was conducted twice. Similarity between the repeated experimental runs was tested by preliminary analyses of variance (ANOVA) using experimental runs as a factor. With no significant differences between the separate experiments (P > 0.05), the data for the experiments were combined, and one-way ANOVA was used for all statistical analyses with the SPSS 13.0 statistical software package (SPSS, Inc., Chicago, IL, USA). Post hoc multiple comparisons were conducted at the 5% level of probability using Duncan’s Multiple Range Test (DMRT).

## Additional Information

**How to cite this article**: Wang, D.-W. *et al*. *Arabidopsis thaliana* as a suitable model host for research on interactions between plant and foliar nematodes, parasites of plant shoot. *Sci. Rep.* **6**, 38286; doi: 10.1038/srep38286 (2016).

**Publisher's note:** Springer Nature remains neutral with regard to jurisdictional claims in published maps and institutional affiliations.

## Supplementary Material

Supplementary File S1

## Figures and Tables

**Table 1:** Effects of *Aphelenchoides besseyi* and *A. ritzemabosi* inoculum concentration and infection time on symptoms severity (mean ± SE, n = 3) of *Arabidopsis thaliana* Col-0 ecotypes.

Treatment		Populations		
Nematode number/per plant	Inoculum times	Ab-S24	Ab-XI	YK
50	10 days	0 f	0 f	0 f
	14 days	0 f	0 f	0 f
	21 days	2.00 ± 0.58 de	2.33 ± 1.33 cde	1.00 ef
	24 days	—	—	3.33 ± 0.88 bcd
100	10 days	1.33 ± 0.33 ef	0 f	0 f
	14 days	2.33 ± 0.33 cde	2.00 ± 1.00 de	0 f
	21 days	5.00 a	4.00 ± 1.00 ab	2.00 ± 0.58 de
	24 days	—	—	4.33 ± 0.33 ab
150	10 days	2.33 ± 0.67 cde	0 f	0 f
	14 days	2.33 ± 0.33 cde	4.33 ± 0.33 ab	0 f
	21 days	5.00 a	5.00 a	3.67 ± 0.88 abc
	24 days	—	—	4.67 ± 0.33 ab

Identical lower case letters indicate means are not significantly different (P > 0.05) for all treatments according to Duncan’s multiple range test. Acording to Zhen *et al*.^24^, the rating of symptom severity on *A. thaliana* caused by foliar nematodes were assigned as follows: rated 0 = no lesion/chlorosis, 1 = 10% lesion/chlorosis, 2 = 11–25% lesion/chlorosis, 3 = 26–50% lesion/chlorosis, 4 = 51–75% lesion/chlorosis, 5 = 75% or more lesion/chlorosis. Ab-S24 and Ab-XI were *A. besseyi*, CFN was *A. ritzemabosi*.
