# Supplementary material for: Arabidopsis thaliana as a suitable model host for research on interactions between plant and foliar nematodes, parasites of plant shoot
Source: Sci Rep. 2016 Dec 2;6:38286. doi: 10.1038/srep38286 (PMC5133616; doi:10.1038/srep38286)
Supplement: Supplementary File S1 [file srep38286-s1.doc]

***Arabidopsis thaliana* as a suitable model host for research on interactions between plant and foliar nematodes, parasites of plant shoot**

# Dong-Wei Wang^¶1^, Xiao-Fang Peng^¶1, 2^, Hui Xie^1*^, Chun-Ling Xu^1^, De-Qiang Cheng^1^, Jun-Yi Li^1^, Wen-Jia Wu^1^, Ke Wang^1^

^1^Laboratory of Plant Nematology and Research Center of Nematodes of Plant Quarantine, Department of Plant Pathology, College of Agriculture, South China Agricultural University, Guangzhou, People’s Republic of China.

^2^Center for Disease Control and Prevention of Guangdong Province, Guangzhou, People’s Republic of China.

**^*^**correspondence and requests for materials should be addressed to H.X. (email: [xiehui@scau.edu.cn](mailto:xiehui@scau.edu.cn))

^¶^these authors contributed equally to this work.


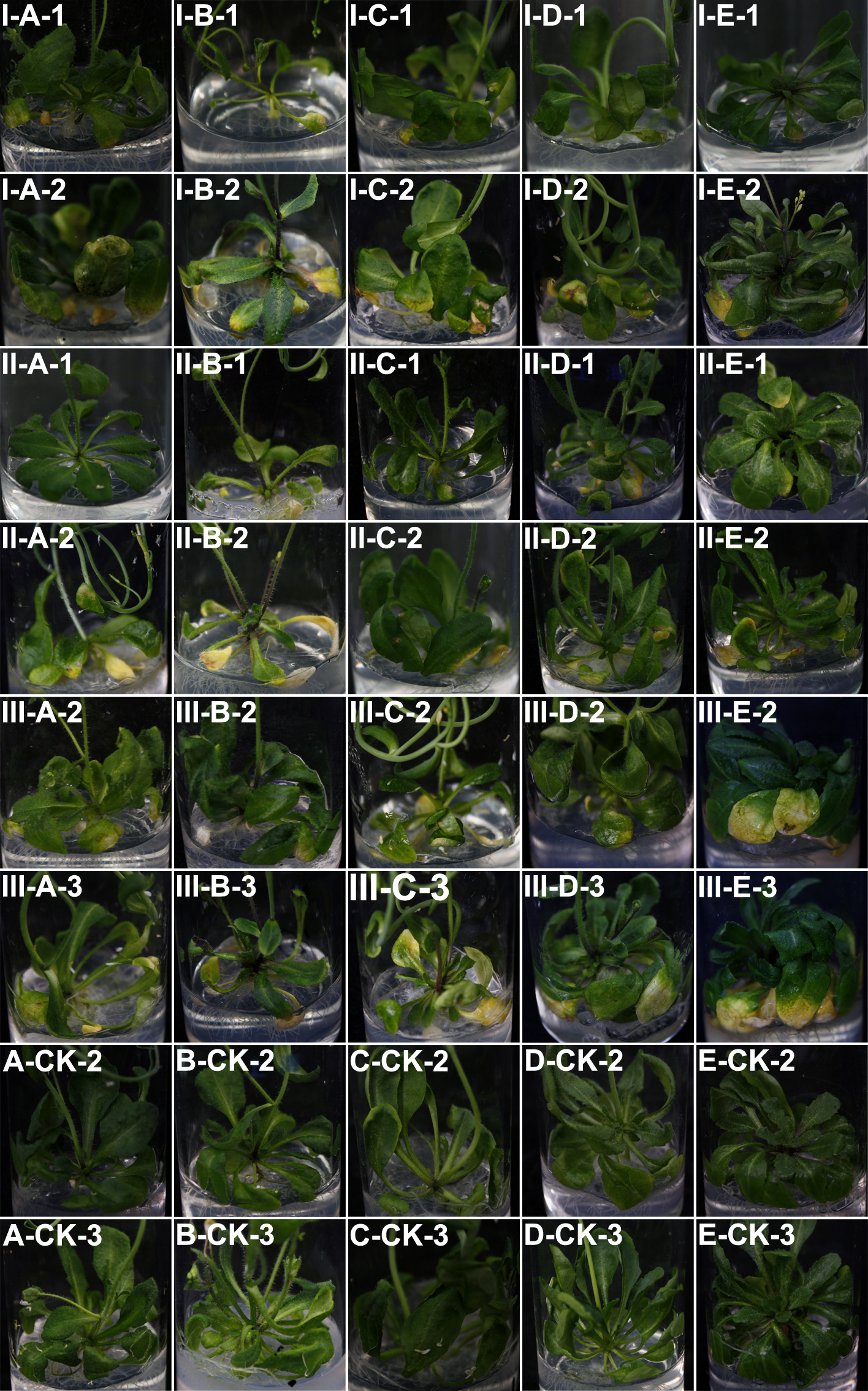


**Figure S1.** Symptoms of five *Arabidopsis thaliana* ecotypes inoculated with 100 nematodes of *Aphelenchoides besseyi* and *A*. *ritzemabosi*. I, II and III: nematodes from Ab-S24 and Ab-XI populations of *A. besseyi* and CFN population of *A*. *ritzemabosi*; A-E: symptoms on leaves of *A. thaliana* Chi, Col-0, Ler, Sha and Ws ecotypes; CK: blank control; 1-3: inoculation times were 14, 21 and 24 days, respectively.


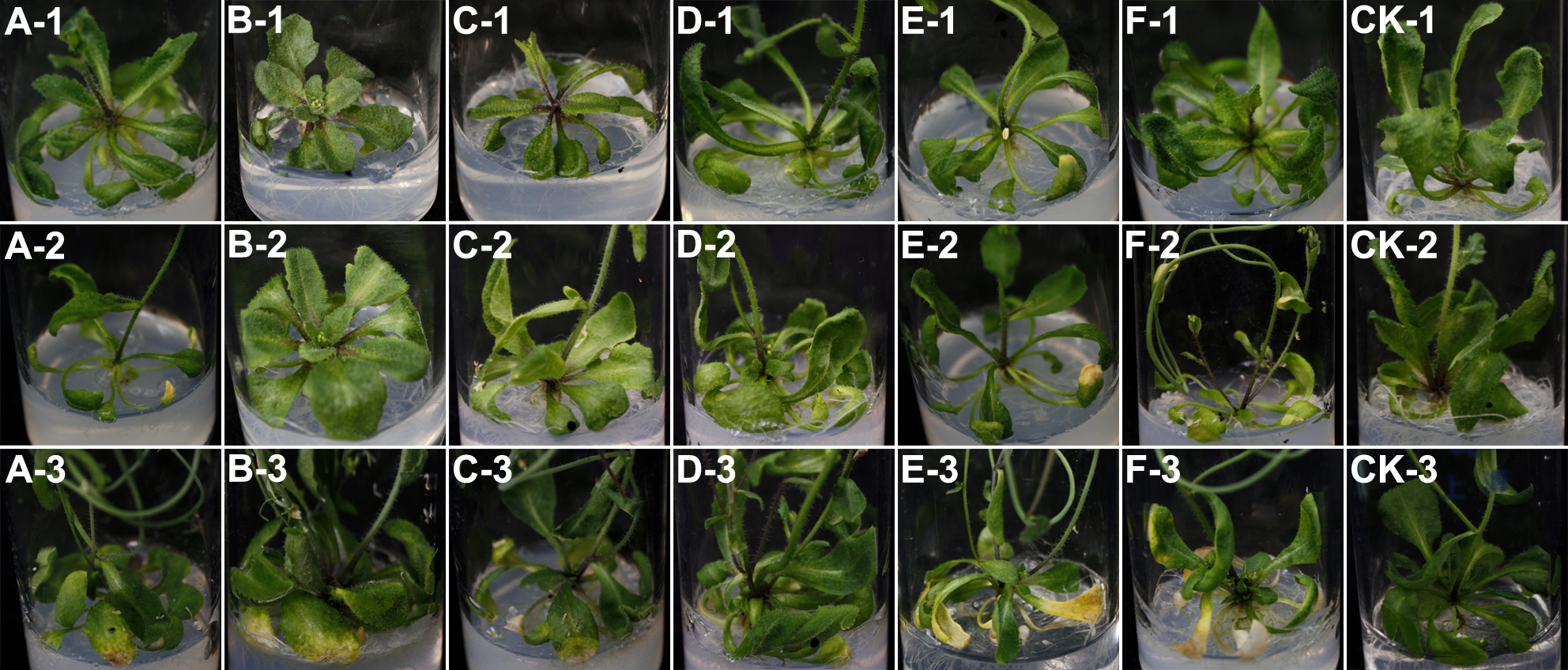


**Figure S2.** Symptoms of *Arabidopsis thaliana* Col-0 ecotype inoculated with 100 nematodes from six different *Aphelenchoides besseyi* populations for 10-21 days. A-F: nematodes from Ab-HB, Ab-HC6, Ab-HN2, Ab-N10, Ab-S24 and Ab-XI populations of *A. besseyi*; CK: blank control; 1-3: inoculation times were 10, 14 and 21 d, respectively.

**Table S1.** Populations of *Aphelenchoides besseyi* and *A. ritzemabosi* used in this study

| Species | Population | Host | Geographical origin |
| --- | --- | --- | --- |
| *A. besseyi* | Ab-HN2 | *Oryza sativa* | Luoshan, Henan |
| *A. besseyi* | Ab-HC6 | *O. sativa* | Huizhou, Guangdong |
| *A. besseyi* | Ab-S24 | *Fragaria ananassa* | Shenzhen, Guangdong |
| *A. besseyi* | Ab-HB | *O. sativa* | Haikou, Hainan |
| *A. besseyi* | Ab-XI | *O. sativa* | Xiamen, Fujian |
| *A. besseyi* | Ab-N10 | *O. sativa* | Nanjing, Jiangsu |
| *A. ritzemabosi* | CFN | *Chrysanthemum* sp. | Kunming, Yunnan |
